# Grain Yield, Starch Content and Activities of Key Enzymes of Waxy and Non-waxy Wheat (*Triticum aestivum* L.)

**DOI:** 10.1038/s41598-018-22587-0

**Published:** 2018-03-14

**Authors:** Yan Zi, Jinfeng Ding, Jianmin Song, Gavin Humphreys, Yongxin Peng, Chunyan Li, Xinkai Zhu, Wenshan Guo

**Affiliations:** 1 Jiangsu Key Lab. of Crop Genetics and Physiology/Co-Innovation Center for Modern Production Technology of Grain Crops/Wheat Research Institute, Yangzhou Univ., Yangzhou, 225009 China; 2 Crop Research Institute, Shandong Academy of Agricultural Sciences, Jinan, 250100 China; 3 Agriculture and Agri-Food Canada, Ottawa Research and Development Centre, K.W. Neatby Building, 960 Carling Avenue, Ottawa, ON K1A 06C Canada

## Abstract

Waxy wheat has unique end-use properties; however, its production is limited due mainly to its low grain yield compared with non-waxy wheat. In order to increase its grain yield, it is critical to understand the eco-physiological differences in grain filling between the waxy and non-waxy wheat. In this study, two waxy wheat and two non-waxy wheat cultivars were used to investigate the differences in starch-associated enzymes processes, sucrose and starch dynamics, yield components, and the final grain yield. The results indicated that the mean total grain starch and amylose content, the average 1000-kernel weight and grain yield of the waxy wheat were lower than those of the non-waxy wheat at maturity. The amylose content was significantly and positively correlated with the activity of GBSS (r = 0.80, *p* < 0.01). Significant positive correlation also exists among activities of AGPase, SSS, GBSS, and SBE, except for GBSS-SBE. In summary, our study has revealed that the reduced conversion of sucrose to starch in the late grain filling stage is the main cause for the low kernel weight and total starch accumulation of the waxy wheat. The reduced conversion also appears to be a factor contributing to the lower grain yield of the waxy wheat.

## Introduction

With the growing in health-conscious consumers, there has been an increase in demand for high-quality wheat flour. The endosperm starch content and composition are two critical parameters for evaluating the quality of wheat flour. For conventionally cultivated wheat, namely non-waxy wheat, starch is a mixture of about 75% amylopectin and 25% amylose. To further improve the quality of wheat flour products such as reducing staling in flour products, keeping baking goods fresh for a longer period of time, and improving the palatability of noodles, the replacement of regular wheat flour with waxy wheat flour may provide a promising solution, as waxy wheat grains contain almost 100% amylopectin in their starch^1^. Common wheat cultivars have three homeologous waxy genes, *Wx-A1*, *Wx-B1* and *Wx-D1*, and waxy wheat mutants lack all three Wx proteins. The Wx proteins are also known as granule-bound starch synthases, which have a role in the synthesis of amylose^1–5^. However, due to low grain yield, the waxy wheat has not been widely cultivated in recent years. To improve the current situation, there is an urgent need for improved understanding of the differences in yield performance between the waxy and non-waxy wheat cultivars at the field level.

The endosperm starch content (approximate 70% of the dry weight) not only influences the grain weight and quality^6,7^, but also reflects the capacity of the sink tissues. Regarding the source-sink relationship, the photoassimilates produced in source tissues as leaf, stem and root were transported into the amyloplast in the form of sucrose, which is the main carbohydrate transported in higher plants. Sucrose is degraded to fructose and uridine diphosphate glucose (UDPG) which are the main precursors of starch synthesis by sucrose synthase (SUS: EC 2.4.1.13)^8,9^. The ability of the sink tissues to accept and convert photoassimilates can be affected by the sink strength, which is an important limiting factor to wheat grain yield, and the activity of SUS can be considered as an indicator of sink strength^8,10,11^.

A coordinated series of enzyme-catalyzed reactions in wheat endosperm result in starch synthesis^12^, including ADPG pyrophosphorylase (AGPase: EC 2.7.7.27), starch branching enzyme (SBE: EC 2.4.1.18), starch debranching enzyme (DBE), granule bound starch synthase (GBSS: EC 2.4.1.21) and soluble starch synthase(SSS: EC 2.4.1.21). GBSS and SSS are the two forms of starch synthase^13,14^. For catalyzing the first unique step in starch synthesis, AGPase is the rate-limiting enzyme and is the most important determinant of seed sink strength^15,16^. Starch synthases catalyse the elongation of the linear glucan chains^13,17^, moreover the different genetic characterization assigns preferential functions for individual isoforms to synthesize amylose or amylopectin. It is generally known that in the endosperm, amylose is synthesized by AGPase and GBSS^13^. SBE that was formerly known as Q-enzyme plays an important role on amylopectin synthesis, which is the only plant enzyme that can introduce α-1,6-glucosidic linkages into α-polyglucans^18,19^.

Previous researches on waxy wheat have focused on the end-use quality, composition of the grain and the expression of the *Wx* genes^4,20–22^. It is reported that the seed weight of inferior spikelets of rice can be improved by increasing the starch-synthesizing enzymes activities^23^. In this study, we compared two waxy wheat cultivars with two non-waxy wheat cultivars to investigate the starch content and accumulation, the activities of enzymes responsible for starch biosynthesis in developing grain, 1000-kernel weight and grain yield with the objective to better understand the differences between the wheat varieties and elucidate factors affecting the 1000-kernel weight and grain yield of the waxy wheat.

## Materials and Methods

### Experimental site

**Figure 1:**
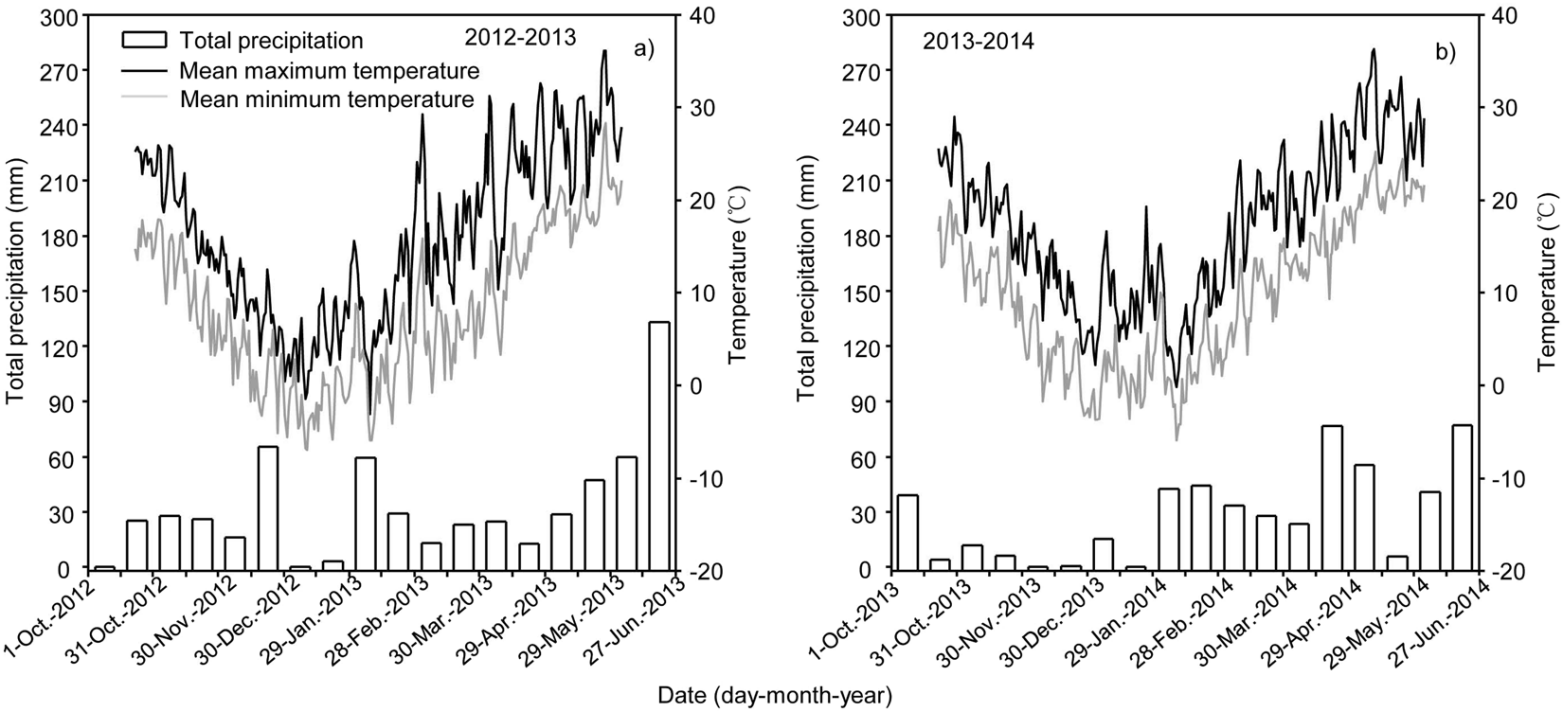
Mean maximum temperature, mean minimum temperature and total precipitate during the wheat growing season of wheat in 2012–2013 (**a**) and 2013–2014 (**b**).

Field experiments were conducted at the Agricultural Experiment Station (32°39′N, 119°25′E) of the Agricultural College of Yangzhou University in China during the winter wheat growing seasons in 2012–2013, and 2013–2014. The field site is situated in a humid subtropical climate zone, with average air temperature of 13 °C to 16 °C, total precipitation of 800 to 1200 mm, total sunshine of 2000 to 2600 h, and a frost-free season of 220 to 240 d. The meteorological data during the wheat growing seasons across the two study years, including temperature and precipitation are shown in Fig. 1. The soil of the site is loamy clay. Pre-planting soil samples were collected prior to any fertilizer applications. The soil before sowing contained 8.38 g kg^−1^ organic matter, 100.01 mg kg^−1^ alkali hydrolysable N, 50.11 mg kg^−1^ Olsen-P, and 149.08 mg kg^−1^ exchangeable K at 0–20 cm soil depth in 2012, while 19.57 g kg^−1^ organic matter, 107.97 mg kg^−1^ alkali hydrolysable N, 33.92 mg kg^−1^ Olsen-P, and 124.23 mg kg^−1^ exchangeable K at 0–20 cm soil depth in 2013.

### Materials

A total of four wheat cultivars were tested, including two types of waxy wheat cultivars Yangnuomai1 (YN1), Ningnuomai1 (NN1), [which lack functional *Wx-A1, Wx-B1, Wx-C1 alleles based on a PCR test (data not shown)*] and two non-waxy wheat cultivars Yangmai20 (Y20) and Yangfumai4 (YF4), (contained three functional *Wx* genes).

### Crop management

All plots were supplied with 240 kg N ha^−1^, applied with the ratio 5:1:2:2 at four stages: pre-sowing, four-leaf stage, jointing stage and booting stage, respectively. Application rate of P_2_O_5_ (P) and K_2_O (K) were both 144 kg ha^−1^: half the amounts of P and K were applied before sowing and the remaining half were applied at the jointing stage. The seeds were sown at a planting density of 225 × 10^4^ ha^−1^ with 30 cm row spacing on 3 November of 2012 and 28 October of 2013. Seedling number was maintained at 225 × 10^4^ ha^−1^ by removing or adding seedlings at the three-leaf stage. Plants were harvested on 3 June of 2013 and 28 May of 2014. Weeds, insects and diseases were controlled as required to avoid yielding loss; other production management practices were based on those used by local farmers.

### Sampling and measurements

#### Measurement of grain yield, components and grain volume

At maturity, an area of 1.2 × 1 m (4 rows included) was harvested manually for the determination of the grain yield. Grain yield was adjusted to 13% moisture. Number of spikes was counted manually for two rows within 1 m. The number of kernels per spike was counted manually to calculate the mean value from 50 continuous spikes. For 1000-kernel weight, 1000 kernels were randomly selected and weighed. The average seed volume was determined via the displacement method^24^.

#### Measurement of the starch, amylose, and amylopectin content

The total starch, amylose, and amylopectin contents were determined via the dual wavelength iodine binding method^25,26^. Wheat grains that were marked flowering on the same day were first ground using a mortar, and the powder was then degreased twice with anhydrous ether. A 100 mg fraction of each sample was used to determine amylose and amylopectin contents. A calibration curve was derived using pure amylose from potato (A0512; Sigma–Aldrich, St. Louis, MO, USA) and pure amylopectin from potato (A8515; Sigma–Aldrich). The sum of amylose and amylopectin contents was designated as the total starch content.

#### Measurement of the activities of AGPase, GBSS, SBE and SSS

Twenty kernels weighed at different filling stages were tested. AGPase activity was assayed according to Nakamura and Cheng^27,28^. SSS and GBSS activities were assayed according to Nakamura and Cheng^27,28^. SBE activity was assayed according to Li *et al*.^29^.

#### Measurement of the sucrose content

The sucrose content was measured by the resorcinol technique^30,31^. Ten dried spikes those were flowered on the same day were selected and killed at 105 °C for 30 min and then dried at 80 °C for measurements of grain weight and starch content. And then they were weighed, and then the dried endosperm was ground into powder for analysis. Samples of the powdered endosperm prepared as mentioned above (100 mg) were extracted with 8 mL of 80% ethanol at 80 °C for 30 min, followed by two extractions with 8 mL of 80% ethanol. The supernatants were combined and purified by 10 g activated carbon overnight, fixed to a constant volume at 50 ml, and purified by filtering. One-hundred microliters of 2 M sodium hydroxide was added to 0.9 ml filtrate, boiled at 100 °C for 10 min. The mixture was cooled to room temperature, then 1 ml of 0.1% resorcinol and 3 ml of 10 mol/L hydrochloric acid were added. The reaction mixture was boiled at 80 °C for 60 min, cooled to room temperature, and the sucrose content was determined by measuring OD at 500 nm.

#### Measurement of the activity of Sucrose Synthase (SUS)

The SUS activity was assayed according to Rufty, Doehlert and Wardlaw^32–34^.

### Statistical analysis

All data were checked for normality based on the Kolmogorov–Smirnov test with SAS proc mixed (SAS V.9.1). The effects of year, cultivar and their interaction on test parameters were tested with year, cultivar as fixed effects, year as repeated measurements, and block as a random effect. Differences among least square means (LSMEANS) for all treatment pairs were tested by the LSD procedure at a significance level of *P* = 0.05. Pearson’s correlation analysis (SAS V.9.1) was performed to establish the relationships among all measured properties. The ANOVA (α = 0.05) was performed to identify significant differences between treatments, multiple comparisons between means (SAS V.9.1) were made using the least significant difference (LSD0.05) test if the F-tests were significant.

## Results

### Sucrose, total starch, amylose and amylopectin contents in grains among waxy and non-waxy wheat

**Figure 2:**
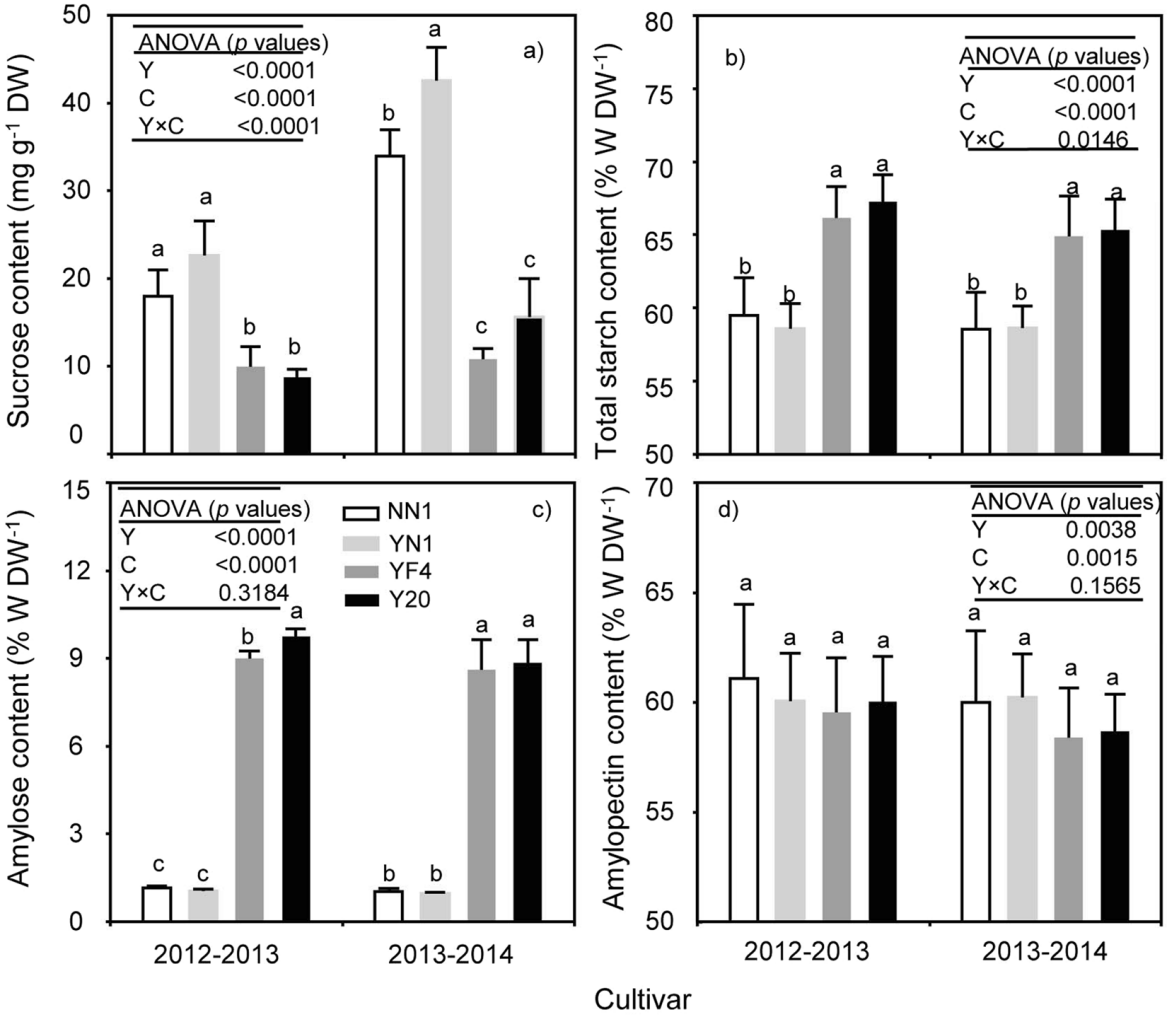
Grain content of sucrose (**a**), total starch (**b**), amylose (**c**) and amylopectin (**d)** in grains between waxy and non-waxy wheats. Values followed by the same letters in each cultivar are not significantly different at *p* < 0.05 level. Each bar represents the SD value.

The test found that grain sucrose content was significantly affected by cultivar, year and their interactions (Fig. 2a). Averaged to cultivars, the sucrose content of waxy wheat was 117.67% and 189.82% higher than that of non-waxy wheat (2012–13 and 2013–14, respectively). Similarly, total starch content was strongly governed by cultivar, year and their interactions (Fig. 2b). The waxy wheat had 14.13% and 12.41% lower total starch content in the 2012–13 and 2013–14 growing seasons respectively, when compared to the non-waxy wheat. Separate effect of year and cultivar significantly affected amylose and amylopectin content, while the interaction of year and cultivar to the amylase and amylopectin content was not significant (Fig. 2c,d). The average amylose contents under waxy wheat were 88.09% and 88.46% lower than that in non-waxy wheat in 2012–13 and 2013–14 seasons respectively. No significant differences in amylopectin levels were detected between waxy and non-waxy wheat cultivars.

### Changes in total starch, amylose, and amylopectin accumulation and accumulation rate in grains of waxy and non-waxy wheat

**Figure 3:**
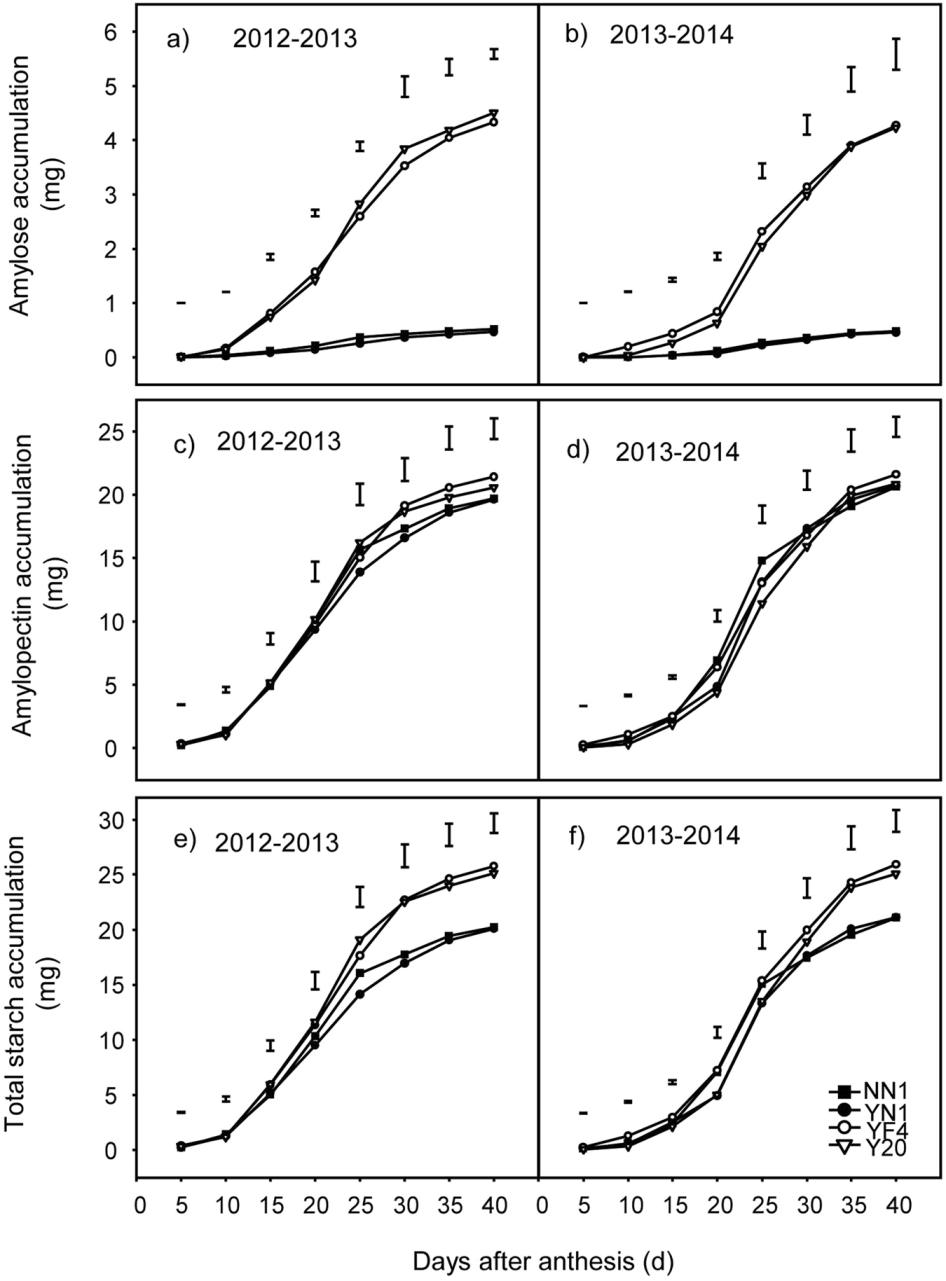
Changes in grain accumulation of amylose (**a**,**b**), amylopectin (**c**,**d**) and total starch (**e**,**f**) in one kernel between waxy and non-waxy wheats after anthesis. Each bar represents the LSD value at *p* < 0.05.

**Figure 4:**
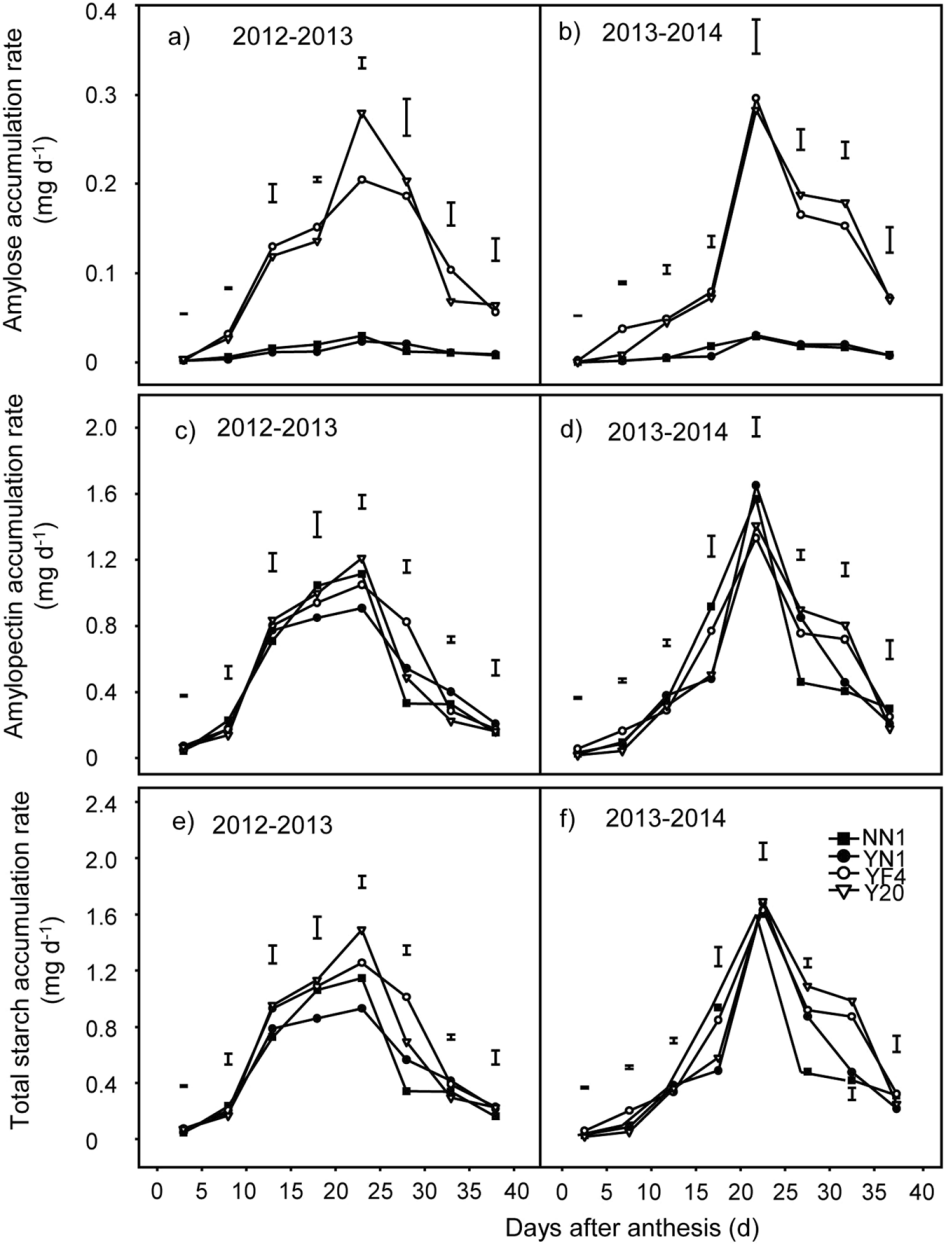
Changes in the accumulation rate of amylose (**a**,**b**), the amylopectin (**c**,**d**) and total starch (**e**,**f**) in one kernel between waxy and non-waxy wheats after anthesis. Each bar represents the LSD value at *p* < 0.05.

As shown in Fig. 3, the grain total starch, amylose, and amylopectin accumulation in waxy and non-waxy wheat showed overall increases during the grain-filling stage in both test years. The total starch (Fig. 3e,f) and amylose accumulation (Fig. 3a,b) were generally lower in waxy wheat compared with non-waxy wheat, particularly after day 25 after anthesis. There was no significant difference observed on amylopectin accumulation between the waxy and non-waxy wheat over both years (Fig. 3c,d). The greatest rates of accumulation for grain total starch, amylose, and amylopectin occurred 10^th^–25^th^ days after anthesis, but decreased after the 25^th^ day after anthesis for both types of wheat (Fig. 4). The amylose accumulation rate in waxy wheat was significantly lower than that in non-waxy wheat (Fig. 4a,b). At 25 days after anthesis, the accumulation rate of total starch in waxy wheat was lower than that in non-waxy wheat (Fig. 4e,f). No significant difference was observed in amylopectin accumulation rate between waxy and non-waxy wheat (Fig. 4c,d). At maturity, the rates of accumulation for total starch, amylose, and amylopectin declined and the accumulation of total starch, amylose, and amylopectin reached the maximum levels.

### Changes in the sucrose content and SUS activity of grains in waxy and non-waxy wheat

**Figure 5:**
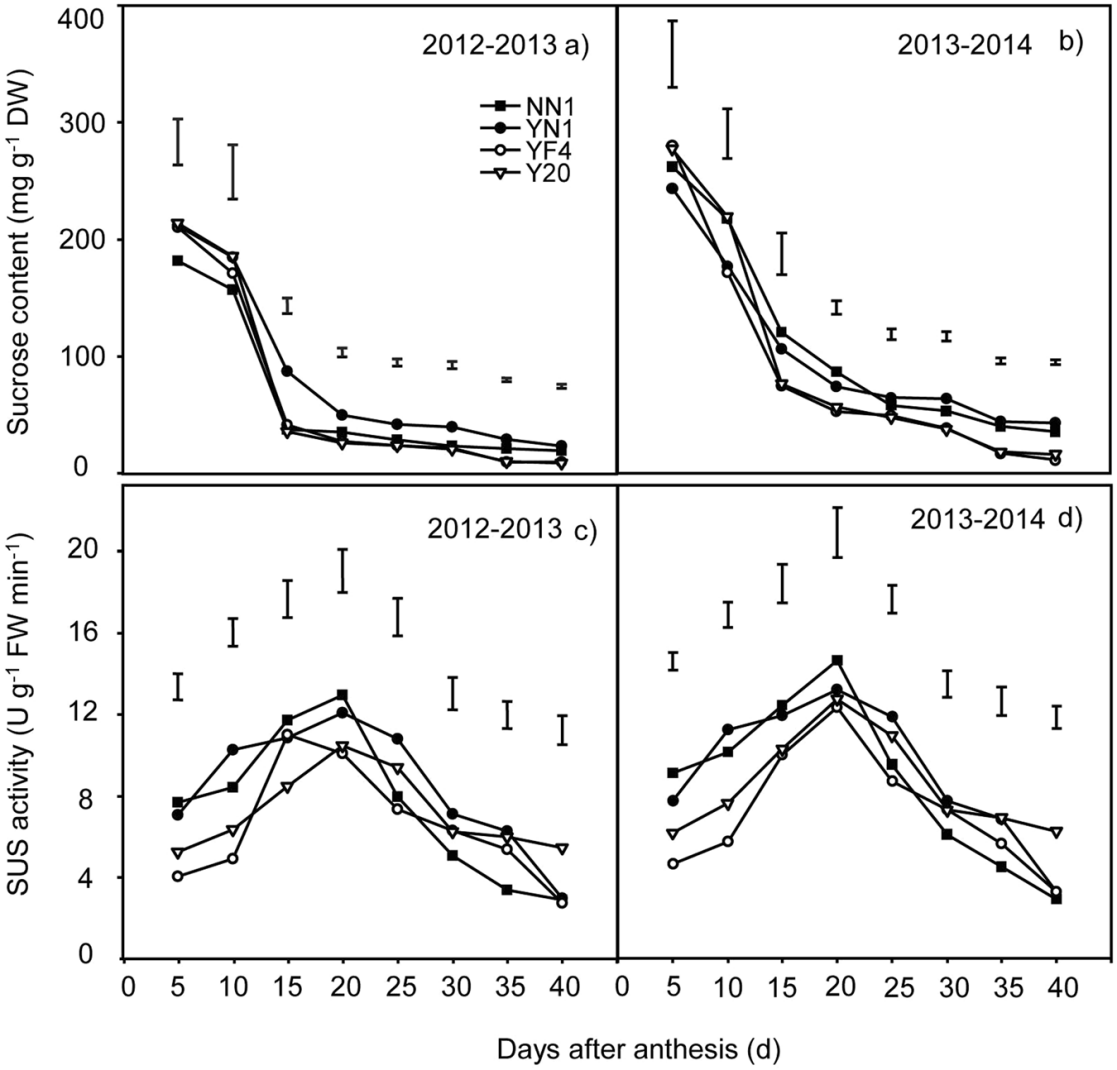
Changes in sucrose content (**a**), and SUS activity in grains between waxy and non-waxy wheats after anthesis. Each bar represents the LSD value at *p* < 0.05.

The grain sucrose content of both waxy and non-waxy wheats declined gradually after anthesis during both growing seasons (Fig. 5a,b). The grain sucrose content of waxy wheat tended to be higher than that of non-waxy wheat beginning ten days after anthesis. As shown in Fig. 5c and d, the SUS activities of the waxy wheat cultivars were higher than that of the non-waxy wheat cultivars up to day 20. Afterwards, the SUS activities of all cultivars dropped

### Changes in the AGPase, GBSS, SSS and SBE activities of grains in waxy and non-waxy wheat

**Figure 6:**
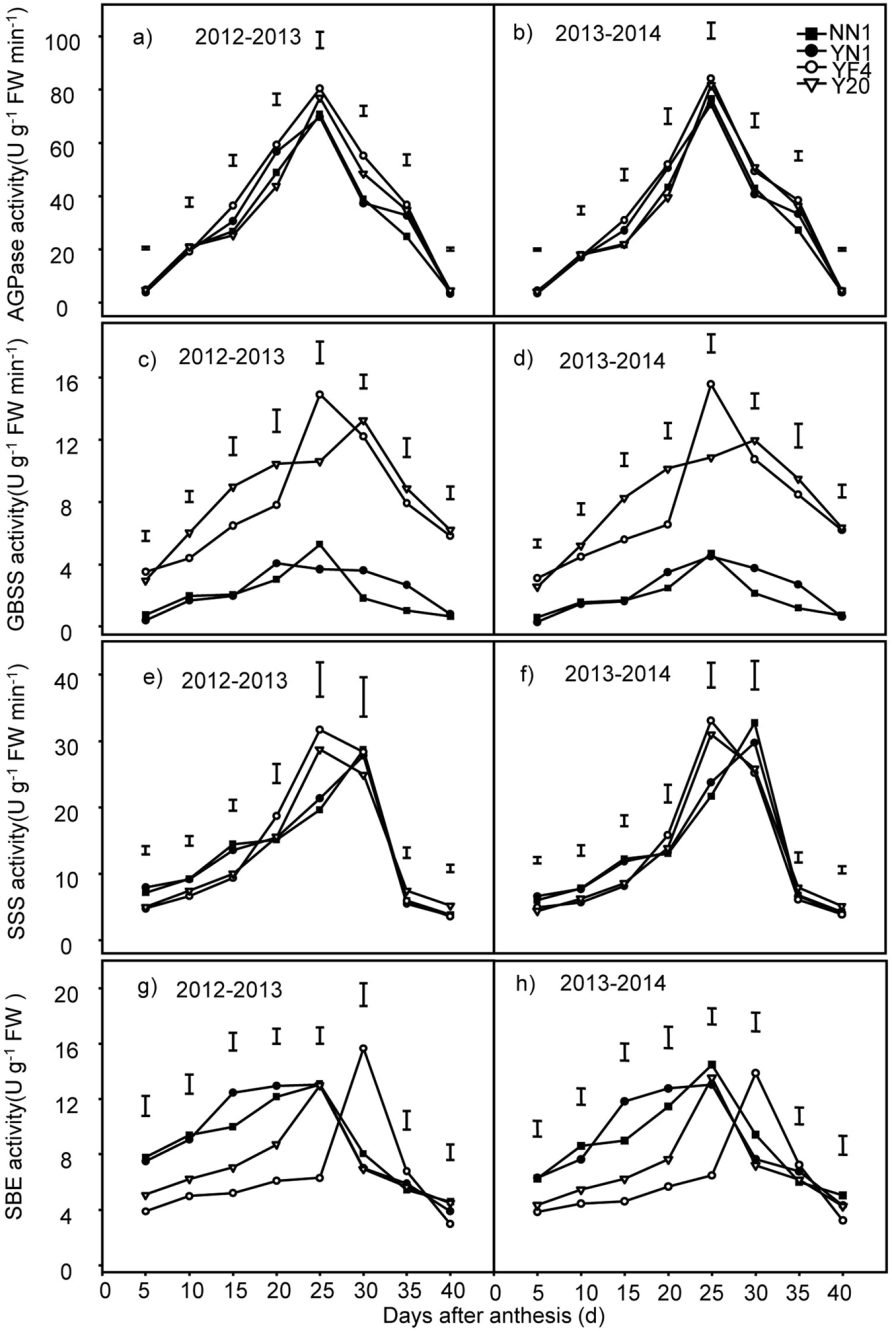
Changes of AGPase (**a**,**b**), GBSS (**c**,**d**), SSS (**e**,**f**) and SBE (**g**,**h**) activity in wheat grains between waxy and non-waxy wheats. Each bar represents the LSD value at *p* < 0.05.

For both the waxy and non-waxy wheat cultivars over both years, the activities of four major enzymes (AGPase, GBSS, SBE and SSS) increased during early grain filling period and decreased during the late period after anthesis. The peak activities of the AGPase and GBSS occurred on the 25^th^ day after anthesis; while for SBE and SSS, the peak activities happened during the period of the 25^th^ to 30^th^ day (Fig. 6). The AGPase in waxy wheat were significantly lower than those in non-waxy wheat, especially after the 20^th^ day after anthesis. Throughout the grain filling, the GBSS activities in waxy wheat were significantly lower than those in non-waxy wheat. In contrast, the activity for SBE was higher in waxy wheat than that in non-waxy wheat before the 25^th^ day after anthesis; this trend was reversed after the 25^th^ day after anthesis. The SSS activity was higher in waxy wheat than that in non-waxy wheat between the 20^th^ and the 30^th^ day after anthesis.

### Grain yield, components and grain volume

The year and cultivar significantly affected the number of kernels per spike (*p*_y_ = 0.0072, *p*_c_ < 0.0001), number of spikes (*p*_y_ = 0.0006, *p*_c_ = 0.0007) and the 1000-kernel weight (*p*_y_ < 0.0001, *p*_c_ < 0.0001); however, the interaction between year and cultivar was not significant. For the grain yield, a significant cultivar effect was detected (*p*_c_ < 0.0001), but not for year and year-cultivar interaction.

**Figure 7:**
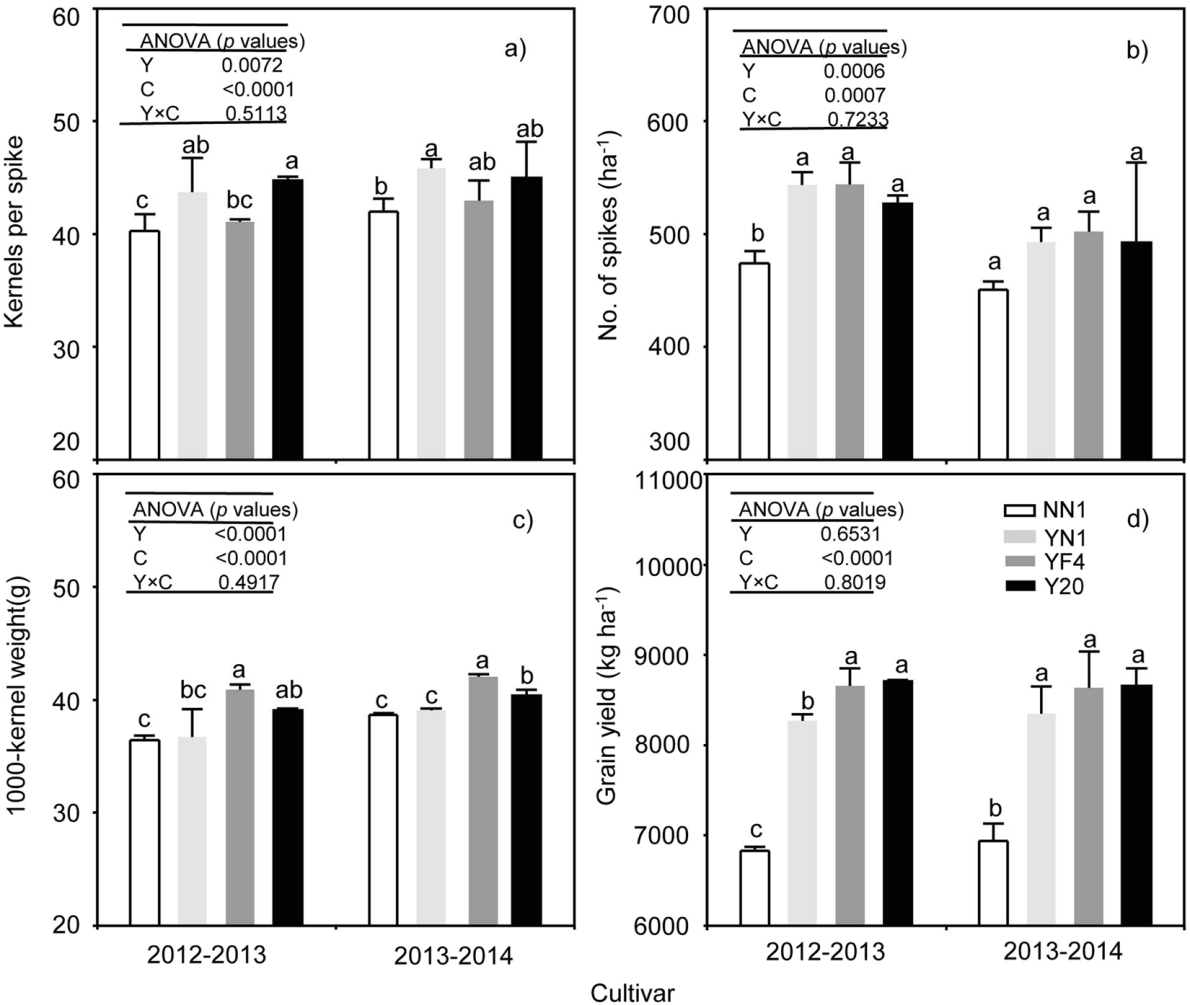
Kernels per spike (**a**), Number of spikes (**b**), 1000-kernel weight (**c**) and grain yield (**d**) between waxy and non-waxy wheats. Values followed by the same letters in each cultivar are not significantly different at *p* < 0.05 level. Each bar represents the LSD value at *p* < 0.05.

**Figure 8:**
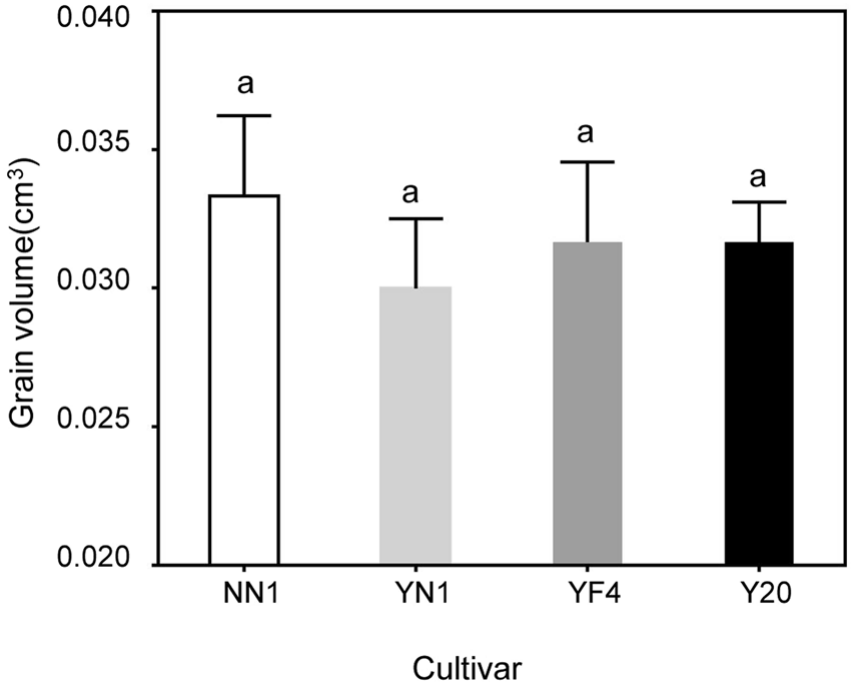
Grain volume between waxy and non-waxy wheats. Values followed by the same letters in each cultivar are not significantly different at *p* < 0.05 level.

The number of kernels per spike in YN1 and Y20 were higher than that in NN1 and YF4 for both test years (Fig. 7a). The lowest value of spike number was observed in NN1 in two years, but there was no significant difference between cultivars in 2013–2014 (Fig. 7b). The 1000-kernel weight and grain yield in waxy wheat were lower than those in non-waxy wheat and in 2013–2014 the difference was significant (Fig. 7c,d). Across cultivars, the kernels per spike, number of spikes, 1000-kernel weight and grain yields of waxy wheat were reduced by 2.22, 5.03, 8.45, 13.02% and 0.11, 5.15, 5.70, 11.62% respectively in 2012–13 and 2013–14 growing seasons, compared with the non-waxy wheat. Trying to verify our hypothesis, we test the grain volume among wheat cultivars in the 2013–2014season,and we found there was no significant difference among wheat cultivars(Fig. 8).

### Correlation analysis

The total starch content in grains was not correlated with the 1000-kernel weight, but amylose was significantly and positively correlated with the 1000-kernel weight (Table 1). Amylopectin and sucrose content did not significantly correlate with the 1000-kernel weight. The total starch and amylopectin accumulation rate were significantly and positively linked with the activities of all five enzymes (r > 0.4, *p* < 0.01), while the amylose content did not significantly correlate with other enzyme activities except for GBSS (Table 2). The total starch content, amylopectin content and amylose accumulation rate were significantly and positively correlated with AGPase, SSS and GBSS activities. In addition, significant correlations were detected between the four enzymatic activities (i.e. AGPase, SSS, GBSS and SBE), with the exception of GBSS-SBE (Table 3).

**Table 1:** Pearson Correlation among 1000-kernel weight and contents of sucrose, total starch and starch components in wheat grains.

	1000-kernel weight	
	r value	*p* value
Sucrose	−0.35	0.40
Total starch	0.69	0.06
Amylose	0.75	0.03
Amylopectin	−0.63	0.09

**Table 2:** Pearson Correlation analysis on total starch accumulation rate, total starch accumulation with the enzyme activities in grains

	Total starch content	Amylose content	Amylopectin content	Total starch accumulation rate	Amylose accumulation rate	Amylopectin accumulation rate
SUS	−0.26^*^	−0.21^ns^	−0.24^ns^	0.43^**^	0.06^ns^	0.47^**^
AGPase	0.43^**^	0.24^ns^	0.43^**^	0.90^**^	0.60^**^	0.90^**^
SSS	0.36^**^	0.18^ns^	0.37^**^	0.73^**^	0.55^**^	0.72^**^
GBSS	0.48^**^	0.80^**^	0.35^**^	0.61^**^	0.90^**^	0.51^**^
SBE	0.08^ns^	−0.13^ns^	0.12^ns^	0.60^**^	0.16^ns^	0.65^**^

^*^Significant at *p* < 0.05.

^**^Significant at *p* < 0.01.

^ns^Not Significant.

**Table 3:** Pearson Correlation analysis among activities of the enzyme in grains.

	AGPase	SSS	GBSS	SBE
AGPase	1			
SSS	0.82**	1		
GBSS	0.59**	0.51**	1	
SBE	0.66**	0.59**	0.11ns	1

^*^Significant at *p* < 0.05.

^**^Significant at *p* < 0.01.

^ns^Not Significant.

## Discussion

### Grain yield an sink strength

The inherent low grain yield of the waxy wheat was reported in previous study^35,36^, and it has limited its commercial production. Improving the agronomic performance is critical in order to meet human demands on high-quality wheat products. In practice, grain yield is determined not only by factors such as the number of spikes, number of kernels per spike and 1000-kernel weight but also by the source-sink relationships^8^. Two key stages, enlargement and grain filling, are responsible for seed size and weight^12,37^.

The sink strength is formed by sink size (i.e. endosperm cell numbers) and sink activity as indicated by activities of the enzymes such as SUS and AGPase in endosperm, which is closely associated with carbohydrate utilization and storage^38^. Gleadow *et al*.^39^ reported that the endosperm cell number was positively correlated with grain size. Sucrose is the main photoassimilate transported to sink tissues, and then the degradation from sucrose to materials for starch synthesis reflects the sink strength in grains^40^. Previous studies have shown that the grain dry weight is potentially determined by sink strength in wheat^39^ and rice^38^. Enhancement of the sink size and sink activity are two critical factors for increasing sink strength. In this study, there was no significant difference in grain volume between the waxy and non-waxy wheat. However, the 1000-kernel weight of waxy wheat was significantly lower than that of non-waxy wheat.

### Sucrose content and SUS activity

The conversion of sucrose to starch in wheat grain was closely related to starch accumulation, for example, high starch accumulation was observed when the conversion of sucrose to starch was high^41^. Moreover, early studies have found that waxy wheats generally have higher sucrose content and lower transforming ability compared with non-waxy wheat^42^. The sucrose content is significantly correlated with the starch synthesis in the early period of grain filling (before 25^th^ days after anthesis) in wheat^43^ and rice^44^. The SUS plays an important role in sucrose synthesis and degradation in plants, especially in wheat grains; It is generally believed that the main function of the SUS is to catalyze the degradation of sucrose in grains^45,46^. The decrease of SUS activity leads to lower rate of conversion from sucrose to starch and a high residual sucrose content^43^, and further resulting in a lower dry matter accumulation characteristic of rice grains^47,48^. In this study, we recorded significant differences in the sucrose content and SUS activity in grains among waxy and non-waxy wheat cultivars after anthesis. Throughout the grain filling, the grains of waxy wheat had higher sucrose content, but SUS activity in grains was different between the earlier and later stage after anthesis. Our results suggested that waxy wheat had higher sucrose degradation and bioavailability during early grain filling stages, but was lower at the later stage compared with non-waxy wheat. More sucrose and less grain starch synthesis ability could be characteristic of waxy wheat in comparison to non-waxy wheat especially in the late period after anthesis. These results were in agreement with some previous studies conducted in waxy^20,42^ and non-waxy^43,46^ wheats.

### Starch accumulation and starch-synthesizing enzymes activities

Starch is synthesized and accumulated during the grain-filling process^49^, which is the major factor influencing both grain yield and quality^7,12^. The first unique step of the starch synthesis is catalyzed by AGPase, which is considered to be the rate-limiting enzyme because of the positive correlation between the activity and starch accumulation rate^8,28,50,51^. In this study, AGPase activity increased at the early grain filling stage, leading to increased accumulation of the total starch, amylose and amylopectin, as well as their accumulation rate. We also found that the AGPase activity in waxy wheat was lower than that in non-waxy wheat in the whole grain filling period, resulting in lower total starch content in waxy wheat seeds compared with non-waxy wheat. At 30d after anthesis, the AGPase activity started to decrease, resulting in a reduction in the rate of starch, amylose and amylopectin accumulation. During the later grain filling period, the starch accumulation and accumulation rate of waxy wheat were lower than that of non-waxy wheat. Similar findings have also been reported in previous studies: in other crops higher AGPase activity resulted in higher crop starch biosynthesis and grain yield including maize^52^, rice^53^ and wheat^16,54^.

GBSS is exclusively involved in the synthesis of amylose, where it catalyzes the extension of long glucans within the amylopectin fraction^15,17^. The absence of waxy protein resulting from waxy mutations in wheat and other cereals could lead to amylose-free starch in endosperm, with low activity of GBSS in waxy wheat^1,2,55^. GBSS activity determines amylose content in the endosperm of rice^56^ and wheat^57^. In this study, the GBSS activity of waxy wheat was significantly lower than that of non-waxy wheat throughout the grain filling period; the amylose accumulation and accumulation rate in waxy wheat were also lower than that in non-waxy wheat. These results indicated that waxy wheat had lower grain starch and amylose synthesis capacity compared with non-waxy wheat, particularly in the case of amylose. Moreover, GBSS is a critical component in the process of amylose formation^58^; some studies have also reported that reduction of amylose content and GBSS activity has been observed in many species that lack amylase and all of these specifically lack GBSSI activity^14^. GBSS is a critical enzyme in the process of amylose biosynthesis^58^ and its activity is closely associated with amylose content as observed in many species that lack amylase and GBSS, specifically the GBSSI activity^14^.

The SSS and SBE play an important role in the formation of the branched structure of amylopectin molecules^17,28,59^. Amylose, amylopectin and total starch accumulation rate in wheat grains were significantly and positively correlated with activities of SBE, SSS and GBSS^60^, and a reduction in enzyme activity appears to be the main factor affecting decreased starch synthesis^43^. In this study, enzymatic activities of SSS and SBE were higher in waxy wheat than in non-waxy wheat, while amylopectin accumulation and amylopectin accumulation rate was significantly higher in waxy wheat compared with the non-waxy wheat during early grain filling stages. During the late grain filling stage, activities of both enzymes were lower, while the amylopectin accumulation and accumulation rate decreased more quickly in waxy wheat compared with non-waxy wheat. These results were similar to those of Tan *et al*.^61^ who reported that lower activities of AGPase, SSS, GBSS and SBE and the lower accumulation of amylose, amylopectin and starch in waxy wheat compared with non-waxy wheat and also in lower starch accumulation cultivar had lower enzyme activities in non-waxy wheat^58^.The SBE activity did not significantly influence the amylopectin content but significantly positively related to the amylopectin accumulation rate, it is postulated that SBE might not play an important role in the formation of amylopectin, but to its fine structure modification, it is stated in maize for the unique function of SBEI in modulating the branching pattern in normal starch by decreasing local clustering of amylopectin branch points^62^.

### Relationship between enzymes activity and kernel weight

Waxy wheat was found to have lower starch content and higher sugar content than non-waxy wheat^22,42^, and also lower starch, amylose and amylopectin accumulation and accumulation rate than non-waxy wheat in the grain filling period^42,61^. It is possible that higher soluble sugar content and lower starch content were due to a low conversion efficiency and the absence of GBSS at the period of grain filling because these two factors have been shown to influence grain weight in waxy rice^63^ and in waxy wheat^20^. Higher activities of SBE, APGase, SSS and GBSS are generally attributed to increased kernel weight in wheat, while starch accumulation rate is significantly correlated with activities of SBE, AGPase, SSS and GBSS in both waxy wheat^61^ and non-waxy wheat^41^. Seed sink strength and seed yield would all be expected to be enhanced by increasing the AGPase activity^16^. The activities of AGPase, SSS, GBSS and SUS play important roles in determining individual grain weight in wheat by regulating starch synthesis in grain endosperm^64^. Further, many studies show that there is a coordinating action in starch biosynthesis among these enzymes^60^. The four important enzymes for starch biosynthesis played the important role but not the whole starch biosyntheses net.

In this study, grain yield appeared to be influenced by sucrose and starch factors. The total starch and amylose content at maturity in grains of waxy wheat were 13.29 and 88.27% lower respectively than those of non-waxy wheat. The 1000-kernel weight of waxy wheat was 7.05% lower than non-waxy wheat, and the grain yield of waxy wheat was 12.33% lower than that of non-waxy wheat. Reduced activities of AGPase, SSS, GBSS and SBE and a lower accumulation of amylose, amylopectin and starch were also observed during the later stage of grain filling in waxy wheat compared with non-waxy wheat. The amylose content was significantly and positively correlated with GBSS activity, while amylose was significantly and positively correlated with the 1000-kernel weight. The total starch content and amylopectin content were significantly and positively correlated with AGPase, SSS and SBE activities. Among the activities of AGPase, SSS, GBSS and SBE, there were significant correlations, except between the activities of GBSS and SBE.

## Conclusions

Our study suggests that low AGPase activity could be the cause of low total starch content in waxy wheat grain, and low GBSS activity could be the contributing factor to insufficient amylose content. During the early grain filling period, no significant difference was found in sink size between the waxy and non-waxy wheat, while higher sucrose degradation and bioavailability and activities of SSS and SBE in grains of waxy wheat were found. Low activities of SSS and SBE in waxy wheat are likely the cause of low amylopectin accumulation in the later stage of grain filling. We speculate that the four enzymes, AGPase, SSS, GBSS and SBE, may have a complementary relationship with regards to starch biosynthesis; thus, the absence of GBSS can reduce the activity of the other three enzymes, leading to low total starch content in grains of waxy wheat. The low activity of SUS during later period of grain filling may have caused the reduced degradation of sucrose and the absence of the precursor of starch in grains of waxy wheat. It appears that weakened starch synthesis in the later stage of grain filling of waxy wheat is likely to be the main cause of low kernel weight and total starch accumulation, which ultimately leads to lower grain yield in waxy wheat.
