# Author Correction: Grain Yield, Starch Content and Activities of Key Enzymes of Waxy and Non-waxy Wheat (*Triticum aestivum* L.)

**DOI:** 10.1038/s41598-018-25219-9

**Published:** 2018-05-01

**Authors:** Yan Zi, Jinfeng Ding, Jianmin Song, Gavin Humphreys, Yongxin Peng, Chunyan Li, Xinkai Zhu, Wenshan Guo

**Affiliations:** 1 Jiangsu Key Lab. of Crop Genetics and Physiology/Co-Innovation Center for Modern Production Technology of Grain Crops/Wheat Research Institute, Yangzhou Univ., Yangzhou, 225009 China; 2 Crop Research Institute, Shandong Academy of Agricultural Sciences, Jinan, 250100 China; 3 Agriculture and Agri-Food Canada, Ottawa Research and Development Centre, K.W. Neatby Building, 960 Carling Avenue, Ottawa, ON K1A, 06C Canada

Correction to: *Scientific Reports* 10.1038/s41598-018-22587-0, published online 14 March 2018

This Article contains typographical errors in the Acknowledgements section.

“This study was supported by the grants from the National Key Research and Development Program of China (2016YFD0300406),”

should read:

“This study was supported by the grants from the National Key Research and Development Program of China (2016YFD0300405),”

